# RBP4 in Ageing

**DOI:** 10.3390/biomedicines14020463

**Published:** 2026-02-19

**Authors:** María Paz Nieto-Bona, María García-De Frutos, Adriana Izquierdo-Lahuerta

**Affiliations:** Departamento de Ciencias Básicas de la Salud, Facultad de Cienciasde la Salud, Universidad Rey Juan Carlos, Avda. de Atenas s/n, 28922 Alcorcón, Madrid, Spain; paz.nieto@urjc.es (M.P.N.-B.);

**Keywords:** RBP4, ageing, mechanisms, organs

## Abstract

**Background:** The protein that binds to retinol 4 (RBP4), is a lipocalin-family protein, secreted primarily by the adipose tissue and the liver, and has also been reported to be produced by other tissues, including the kidney. This protein mediates the transport of vitamin A (retinol) in the circulation, bound to a transporter protein, transthyretin. In recent years, RBP4 has been shown to contribute to the development of insulin resistance and a range of metabolic disorders such as type 2 diabetes mellitus, gestational diabetes, obesity, metabolic syndrome, hyperuricaemia, metabolic dysfunction-associated steatotic liver disease (MASLD), and cardiorenal diseases. **Objectives:** The objective was to analyse the role of RBP4 in ageing, as well as its mechanisms and effects across organs and systems. **Results:** Circulating RBP4 levels increase with age and have been related to the onset of various processes like sarcopenia, elevated neurodegenerative markers in the brain, and an increase in TSH levels. Furthermore, it appears that in ageing, the rise in RBP4 is related to the development of atherogenesis, chronic kidney disease, and osteoarthritis. These effects appear to be mediated by chronic inflammation along with the development of insulin resistance, increased oxidative stress and mitochondrial dysfunction, inhibition of autophagy, and intestinal dysbiosis. **Conclusions:** RBP4 is a factor to be taken into account in the ageing process, as it has been shown that elevated circulating serum levels in older individuals lead to and accelerate deterioration across different organs or systems.

## 1. Introduction

Ageing is characterized by a progressive decline in the function of organisms during adulthood. The global population is ageing, driven by increased life expectancy. According to the WHO, by 2030, approximately one out of six people in the world will be aged 60 years or over. By 2050, the world’s population of people aged 60 years and older is expected to double.

From a cellular and molecular perspective, ageing has characteristic hallmarks that generally act together, making them difficult to disentangle. Today, up to twelve of these hallmarks have been proposed [1]: genomic instability, telomere attrition, epigenetic alterations, loss of proteostasis, disabled macroautophagy, deregulated nutrient-sensing, mitochondrial dysfunction, cellular senescence, stem cell exhaustion, altered intercellular communication, chronic inflammation, and dysbiosis.

These cellular and molecular alterations associated with ageing lead to functional deterioration across all organs. However, despite broadly similar trajectories, the progression of the decline is asynchronous both between and within organs [2]. It appears that the faster ageing of a particular organ may increase vulnerability to morbidity affecting multiple organs [3]. This supports the hypothesis that specific inter-organ relationships during ageing contribute to variations in the risk of developing diseases [3].

Furthermore, it has been observed that lifestyle (including diet, occupation, and medication use) is associated with different rates of ageing in different organs [4]. Moreover, sex influences organ function, leading organ trajectories and susceptibility to age-related diseases. This is possibly related to known differences in fat storage, sex hormone regulation, and renal haemodynamics [2]. In this regard, ageing models that predict specific organ/system diseases indicate that men age faster than women [4]. All of these factors cause biological ageing to progress at different rates among individuals.

RBP4 is a 21 k-Da lipocalin-family protein, whose human gene is located in position 10q23.33. First identified in 1968 [5], it is primarily produced by the liver, followed by the adipose tissue and kidneys. It is a secreted protein that transports retinol (vitamin A) through blood plasma. In plasma, the RBP–retinol complex binds to transthyretin (TTR) [6], thereby preventing its loss through renal filtration. TTR, also known as prealbumin, is a homotetrameric transport protein with four binding sites: two for thyroxine and two for the retinol–RBP complex. TTR is responsible for transporting thyroxine (T4) and the retinol–RBP binding complex to different parts of the body [7]. Its classic function is to mediate the transport of retinol to peripheral tissues via the receptor STRA6. Although most of RBP4’s actions depend on its role in retinoid homeostasis, etinol-transport independent functions have been described [6]. In recent years, RBP4 has been shown to contribute to the development of insulin resistance and other metabolic disorders such as type 2 diabetes mellitus, gestational diabetes, obesity, metabolic syndrome, hyperuricaemia, metabolic dysfunction-associated steatotic liver disease (MASLD), hypertension, cardiovascular diseases and chronic kidney disease [6,8,9,10,11,12,13]. Additionally, elevated levels of this protein have been associated with a poorer disease prognosis, supporting its potential utility as both a biomarker and a therapeutic target.

**Figure 1:**
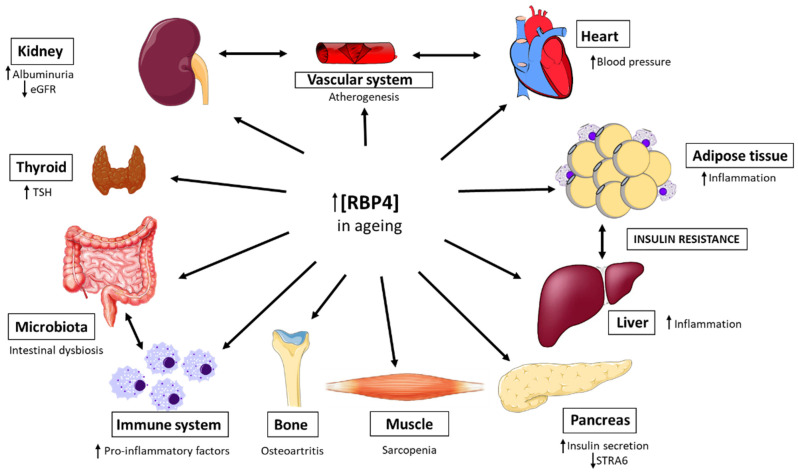
Effects of RBP4 on ageing in different organs and systems. During aging, an increase in the blood concentration of RBP4 is observed. This alteration has been associated with different effects in various organs, such as an increase in pro-inflammatory factors and an activation of macrophages toward the M1 phenotype, which promotes a chronic inflammatory state in white adipose tissue and the liver and the establishment of intestinal dysbiosis. In addition, the pancreas promotes an increase in insulin secretion, which, together with the above effects, leads to the development of insulin resistance. Furthermore, this increase in RBP4 in the blood is related to increased oxidative stress and mitochondrial damage in the vascular endothelium, leading to the development of atherogenesis. The heart is also affected, with reports of hypertension and chronic kidney disease (CKD). Osteoarthritis appears in the bones, muscle mass is lost (sarcopenia), and finally, it has been shown that an increase in RBP4 concentration correlates with an increase in thyroid-stimulating hormone (TSH). STRA6: Signalling receptor and transporter of retinol 6; eGFR: estimated glomerular filtration rate.

In this review, we have compiled evidence of the role of RBP4 in ageing across multiple organs and systems (Figure 1), relating them to the mechanisms and hallmarks of this physiological process. The aim is to understand the contribution of this adipokine to the process.

## 2. Mechanisms of Action of RBP4 in Ageing

### 2.1. Altered Intercellular Communication: Increase in Serum RBP4 Levels

In recent years, studies have linked serum RBP4 levels to age [14,15,16], indicating a possible role in ageing. Although this effect was initially reported in postmenopausal women and was attributed to an increased proportion of adipose tissue, particularly visceral fat, driven by menopause-related dysregulation of lipid metabolism following oestrogen loss, subsequent studies have shown that elevated serum RBP4 levels also rise with age in men [15].

These studies have shown that there is no correlation between RBP4 concentrations in blood and insulin sensitivity, body fat percentage, or dyslipidaemia in older subjects [15]. This suggests that the age-related elevation of RBP4 may be an independent effect, and not merely secondary to other metabolic diseases or comorbidities.

It has also been reported that higher concentrations of RBP4 are associated with neurodegenerative markers of brain ageing in mid-life, independent of cardiometabolic factors [17]. Consistently, RBP4 levels are increased in brain extracts from patients with Alzheimer’s disease compared to controls [18]. This age-related disease is caused by the alteration of protein homeostasis or proteostasis, a hallmark of ageing that leads to the accumulation of misfolded proteins, which aggregate into extracellular amyloid plaques [1].

Furthermore, in elderly patients with osteoarthritis, RBP4 levels in blood positively correlate with those in synovial fluid [19]. However, determining RPB4’s contribution to ageing is complex, as it is often accompanied by metabolic dysfunctions and cellular senescence.

### 2.2. Deregulated Nutrient-Sensing: RBP4 and the Insulin Signaling Pathway

The somatotropic axis, which involves growth hormone (GH), has been linked to ageing. GH, secreted by the pituitary gland, acts on its receptor in hepatocytes, stimulating the secretion of IGF1, which through IGF1R, promotes growth by activating the phosphatidylinositol 3-kinase (PI3K)/protein kinase B (Akt) pathways and the mechanistic target of rapamycin complex-1 (mTORC1) [20]. The use of mTOR complex inhibitors has been shown to extend life in animal models and improve immune response in humans [1].

RBP4 can impair insulin signalling by reducing tyrosine phosphorylation of IRS-1, leading to a decreased activation of the PI3K-Akt pathways [21]. A lack of activation of this pathway prevents glucose transporter 4 (GLUT4) from translocating from the cytoplasm to the plasma membrane and responding to the insulin signal, thereby diminishing glucose uptake. In addition, RBP4 also activates c-Jun N-terminal kinase (JNK) [22] and, in pancreatic beta cells, can bind to the STRA6 receptor, exerting an inhibitory effect on insulin synthesis through the Janus kinase 2 (JAK2)/STAT1/ISL-1 pathway [23].

### 2.3. Chronic Inflammation and Intestinal Dysbiosis: Implications of RBP4

**Figure 2:**
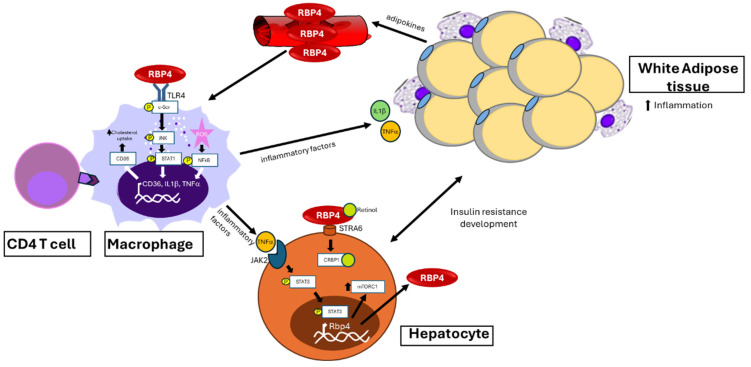
Effects and pathways activated by increased serum levels of RBP4 in immune system cells (macrophages and T cells), hepatocytes, and adipocytes. CD36: cluster of differentiation 36; CRBP1: cellular retinol binding protein 1; c-Scr: cellular-non-receptor tyrosine kinase; IL1β: interleukin 1 beta; JAK2: Janus kinase 2; mTORC-1: mechanistic target of rapamycin complex-1; NF-κB: nuclear factor kappa-light-chain-enhancer of activated B cells; ROS: reactive oxygen species; STAT: signal transducer and activator of transcription; STRA6: signalling receptor and transporter of retinol 6; TLR4: Toll-like receptor 4; TNF-α: tumour necrosis factor-alpha.

Chronic low-grade inflammation is a hallmark of ageing, and thus the term “inflammageing” has been introduced [24]. This process involves the activation of mechanisms like those that occur in chronic inflammation associated with metabolic diseases. In this regard, numerous studies show that RBP4 is a key factor that contributes to adipose tissue inflammation and insulin resistance by promoting crosstalk between the innate and adaptive immune systems [25]. RBP4 stimulates pro-inflammatory cytokines in metabolic diseases that involve the development of insulin resistance, such as tumour necrosis factor-alpha (TNF-α) and interleukins via the Toll-like receptor/JNK pathway. The retinol-RBP4 complex directly inhibits insulin signalling in adipocytes by activating JAK2/STAT5/suppressor of cytokine signalling 3 pathways. This retinol-dependent mechanism requires stimulation of the vitamin A receptor stimulated by retinoic acid (STRA6) [26]. Increased RBP4 appears to elicit stronger inflammation in adipose tissue than in the liver. In adipose tissue, RPB4 directly activates antigen-presenting cells (APCs) in a JNK-dependent manner, induces CD206+ macrophages to secrete proinflammatory cytokines, and promotes the polarization (Th1) and proliferation of CD4 T cells, thereby inducing insulin resistance (Figure 2). In both tissues (adipose and liver), the innate immune system (macrophages) is activated; however, activation of the adaptive immune system (CD4 T cells), which amplifies inflammation, is observed only in visceral adipose tissue [25].

In age-related chronic inflammation, the gut microbiota appears to play a crucial role. Intestinal dysbiosis, an imbalance in the composition and metabolic activity of the gut microbiota, plays a fundamental role in ageing-inflammation by releasing pro-inflammatory microbial products, influencing circadian rhythm, and interacting with other organs and physiological systems [24]. In this context, it appears that RBP4 also contributes to the gut microbial composition, as genotype-dependent differences have been observed between *Rbp4* knockout and wild-type mice [27].

### 2.4. Endothelial Dysfunction and Cardiorenal Disease: The Role of RBP4

Epidemiological studies have reported a positive correlation between elevated RBP4 levels and the prevalence of cardiovascular diseases (CVD) including hypertension (HT) and coronary artery disease (CAD), indicating a fundamental role for RBP4 cardiovascular health [27].

Animal studies have shown that RBP4 upregulation impairs endothelial mitochondrial function and promotes vascular oxidative stress, leading to endothelial dysfunction, a key feature of atherogenesis [28]. Moreover, in middle-aged and older individuals, elevated serum RBP4 levels have been associated with enhanced atherogenesis by promoting macrophage-derived foam cell formation through the activation of CD36-mediated cholesterol uptake via the c-Src-JNK-STAT1 signalling pathway [29].

In recent years, it has been observed that cardiac dysfunction is accompanied by renal dysfunction, with reciprocal effects between the two organs, a condition known as cardiorenal syndrome. In this context, elevated urinary RBP4 levels have been associated with renal pathologies in both humans and animal models, including chronic renal disease, glomerulopathies, diabetic nephropathy, and lipotoxic damage [8,11,30]. RBP4 is a low-molecular-weight protein (21 kDa) that freely filters through the glomeruli and is then almost entirely reabsorbed in the proximal tubules [11]. The appearance of RBP4 in urine is due to an alteration in the glomerular filtration barrier that allows proteins to escape (proteinuria) and is therefore a sign of kidney damage and possibly the development of chronic kidney disease [8,11,30]. Moreover, renal RBP4 levels correlate positively with parathyroid hormone-related protein (PTHrP) [30], a factor associated with kidney disease, cachexia, and adipose tissue browning [31].

### 2.5. Mitochondrial Dysfunction and RBP4 in the Development of Age-Related Sarcopenia

The fibre-type profile of skeletal muscles undergoes marked changes across postnatal development and ageing [32]. RBP4 influences muscle fibre-type distribution by promoting a shift from type 1 (slow oxidative) to type 2 (glycolytic) [33]. This RBP4-induced transition towards type 2 fibres may reduce endurance capacity and increase fatigability. Ageing-related fibre-type remodelling is also accompanied by mitochondrial dysfunction. In this regard, implications of RBP4 in mitochondrial dysfunction in muscle have also been described [28,33,34].

In addition to changes in fibre-type proportions during ageing, skeletal muscle undergoes a progressive loss of mass known as sarcopenia. In older adults higher levels of RBP4 have been associated with an increased risk of sarcopenia, reduced muscle mass, and physical dysfunction. Moreover, serum RBP4 levels correlate positively with sarcopenia severity [35].

Recently, RBP4 has also been shown to promote fat infiltration and muscle atrophy in skeletal muscle via a STRA6-dependent mechanism involving the JAK2/STAT3 pathway [33].

### 2.6. Loss of Metabolic Homeostasis and Circadian Rhythms: Thyroid Dysfunction and RBP4

Thyroid hormones play a fundamental role in maintaining metabolic homeostasis [36]. During ageing, the thyroid undergoes morphological and functional changes, including a reduction in the size of the gland, a decrease in the production of the hormone triiodothyronine (T3), the development of nodules and thyroid dysfunction manifesting as hypothyroidism or hyperthyroidism. Collectively, these alterations can impair thyroid regulation of metabolism, thermogenesis and immunity [37].

The incidence of thyroid disease, particularly hypothyroidism, increases with age. In healthy older populations, thyroid-stimulating hormone (TSH) levels tend to rise and have also been reported to exhibit higher plasma levels of RBP4 than those with normal thyroid function. In addition, TSH levels have been shown to correlate with RBP4 levels in older people with normal glucose tolerance, regardless of the degree of obesity or amount of body fat [38].

Thyroid function is regulated by circadian clocks, which are altered with ageing. Ageing affects all structures and systems in the body, including the circadian system, and is associated with three common features of circadian rhythmicity: loss of amplitude, increased fragmentation, and phase shift, typically phase advance [39]. Indeed, it has been shown that resynchronising circadian rhythms delays ageing [40,41]. Consistently, the *Rbp4* gene has been reported to be under the control of the circadian clock [42].

## 3. Discussion

Ageing is a physiological process marked by a progressive decline of bodily functions [1]. RBP4 is an adipokine implicated in metabolic disorders [6,8,9,10,11,12,13], and elevated circulating levels have increasingly been associated with poorer outcomes across several age-related conditions. In this review, we have analysed the role of RBP4 and its mechanisms of action in different organs and systems during ageing.

However, it remains difficult to determine how this process is initiated and which organ or cell type is primarily responsible for the increased circulating RBP4 levels observed in older individuals without an associated pathology [15]. It is unclear whether the elevation in serum levels of this protein observed with age could act as a signalling factor propagating damage across multiple organs and systems.

Building on these findings, it could be proposed that RBP4 acts as a circulating pro-ageing factor that conveys damage signals from a primary site of dysfunction. White adipose tissue is a leading candidate source given its ageing-associated chronic inflammation [43].

## 4. Conclusions

RBP4 is a factor to be considered in the ageing process, as elevated circulating levels in older individuals have been associated with accelerated functional decline across multiple organs and systems. Age-related increases in RBP4 have been linked with loss of muscle mass (sarcopenia), higher levels of neurodegenerative markers in the brain, and elevated TSH levels. It has also been associated with pro-atherogenic processes, chronic kidney disease, and osteoarthritis. These associations may be mediated through mechanisms of ageing such as chronic inflammation and the development of insulin resistance in adipose tissue and liver, endothelial mitochondrial dysfunction and intestinal dysbiosis.

## 5. Future Directions

Therefore, RBP4 should be considered from two perspectives: first, as a blood biomarker of aging. However, RBP4 should also be considered as a potential therapeutic target, since it is possible that blocking the increase in serum RBP4 levels could slow down the ageing process. If age-related increases in circulating RBP4 contribute to multi-organ damage, strategies aimed at limiting this rise could, in principle, help attenuate age-associated functional decline. However, further work is needed to determine when and how this increase is triggered, and to identify the cell types and tissues primarily responsible for increasing RBP4 in older individuals, without apparent comorbidities or pathologies.

## Data Availability

No new data were created or analyzed in this study. Data sharing is not applicable to this article.

## Abbreviations

The following abbreviations are used in this manuscript:

AKT	Protein kinase B
APCs	Antigen-presenting cells
CD36	Cluster of differentiation 36
CKD	Chronic kidney disease
CRBP1	Cellular retinol binding protein 1
CVD	Cardiovascular diseases
GH	Growth hormone
GLUT4	Glucose transporter 4
eGFR	Estimated glomerular filtration rate
JAK2	Janus kinase 2
JNK	c-Jun N-terminal kinase
MASLD	Metabolic dysfunction-associated steatotic liver disease
mTOR	Mechanistic target of rapamycin
NF-κB	Nuclear factor kappa-light-chain-enhancer of activated B cells
PI3K	Phosphatidylinositol 3-kinase
PTHrP	Parathyroid hormone-related protein
RBP4	Retinol binding protein 4
TLR4	Toll-like receptor 4
TNF-α	Tumour necrosis factor-alpha
TTR	Transthyretin
TSH	Thyroid-stimulating hormone
T3	Hormone triiodothyronine
STAT	Signal transducer and activator of transcription
STRA6	Signalling receptor and transporter of retinol 6
